# The Prognostic Role of Frailty and Its Recognition With Simple FRAIL and Fried Frailty Questionnaires in Advanced Cancer Patients

**DOI:** 10.1002/jcsm.70076

**Published:** 2025-10-12

**Authors:** Pia Weinländer, Sara Hadzibegovic, Jan Porthun, Lucie Kretzler, Ruben Evertz, Alessia Lena, Laura‐Carina Lück, Jonathan L. Hella, Ursula Wilkenshoff, Andrea Stroux, Kalliopi Keramida, Anne Letsch, Dominik Paul Modest, Lars Bullinger, Ulrich Keller, Mahir Karakas, Youssef S. Abdelwahed, Philipp Attanasio, Ursula Rauch, Carsten Skurk, Wolfram Doehner, Ulf Landmesser, Stephan von Haehling, Markus S. Anker

**Affiliations:** ^1^ Charité – University Medicine Berlin Corporate Member of Free University Berlin and Humboldt‐University Berlin Berlin Germany; ^2^ German Centre for Cardiovascular Research (DZHK), Partner Site Berlin Berlin Germany; ^3^ Department of Cardiology, Angiology and Intensive Care Medicine Campus Virchow Clinic German Heart Center Charité Berlin Germany; ^4^ Department of Cardiology, Angiology and Intensive Care Medicine Campus Benjamin Franklin German Heart Center Charité Berlin Germany; ^5^ Norwegian University of Science and Technology, Campus Gjøvik Gjøvik Norway; ^6^ Department of Cardiology and Pneumology University of Göttingen Medical Center Göttingen Germany; ^7^ German Centre for Cardiovascular Research (DZHK), Partner Site Lower Saxony Göttingen Germany; ^8^ Institute of Biometry and Clinical Epidemiology Charité Medical University Berlin Berlin Germany; ^9^ Cardiology Department General Anti‐Cancer Oncological Hospital, Agios Savvas Athens Greece; ^10^ Department of Cardiology, Athens University Hospital Attikon National and Kapodistrian University of Athens Medical School Athens Greece; ^11^ Charité Comprehensive Cancer Center Charité – University Medicine Berlin Berlin Germany; ^12^ Department of Hematology, Oncology and Cancer Immunology Charité – University Medicine Berlin Corporate Member of Free University Berlin and Humboldt University Berlin Berlin Germany; ^13^ German Cancer Consortium (DKTK), Partner Site Berlin Berlin Germany; ^14^ German Cancer Research Center (DKFZ) Heidelberg Germany; ^15^ Max Delbrück Center Berlin Germany; ^16^ German Centre for Cardiovascular Research, Partner Site HH/Kiel/HL Hamburg Germany; ^17^ Department of Intensive Care Medicine University Medical Center Hamburg‐Eppendorf Hamburg Germany; ^18^ Centre for Stroke Research, Berlin Charité‐Universitätsmedizin Berlin Berlin Germany; ^19^ Berlin Institute of Health Center for Regenerative Therapies (BCRT) Charité ‐ Universitätsmedizin Berlin Berlin Germany; ^20^ Deutsches Herzzentrum der Charité, Department of Cardiology, Angiology and Intensive Care Medicine Berlin Germany; ^21^ Berlin Institute of Health Charité – University Medicine Berlin Berlin Germany; ^22^ School of Cardiovascular and Metabolic Health University of Glasgow Glasgow UK

**Keywords:** cancer, frailty, Fried Frailty Phenotype, mortality, Simple FRAIL Questionnaire

## Abstract

**Background:**

Patients with advanced cancer frequently suffer from frailty associated with vulnerability and adverse outcomes. Our aim was to assess the prevalence of frailty and elucidate the utility of two commonly used frailty questionnaires in an advanced cancer population.

**Methods:**

The Fried Frailty Phenotype (FFP) and Simple FRAIL Questionnaire (SFQ) were assessed in hospitalized patients with mostly advanced cancer. Patients were classified by both questionnaires as frail (3–5 points), pre‐frail (1–2 points) and robust (0 points) and followed up for all‐cause mortality. Utility was evaluated with correlation and survival analysis.

**Results:**

From 11/2017 to 02/2020, 251 mostly advanced cancer patients (61 ± 13 years, 53% men, BMI 25.3 ± 4.8 kg/m^2^, 78% cancer stage ≥ 3) were prospectively enrolled. In cancer patients, according to the FFP and SFQ, 17%/13% were frail, 52%/41% prefrail and 31%/47% robust. The correlation between both scores was strong (*r*_*s*_ = 0.65, *p* < 0.001). Both scores were predictors of mortality of cancer patients in univariable and multivariable Cox proportional hazards analyses (multivariable adjusted: per 1 point: FFP: HR 1.36, 95% CI, 1.15–1.61, *p* < 0.001; SFQ: HR 1.29, 95% CI, 1.09–1.52, *p* = 0.003—adjustment for age, cancer stage/type, anti‐cancer therapy naïve, sex, BMI, CKD and anaemia). The time‐dependent multivariable adjusted area under the receiver operating characteristic curve for 6‐/24‐month survival follow‐up for the FFP was 0.78 (95% CI, 0.70–0.86)/0.92 (95% CI, 0.87–0.98) and for the SFQ was 0.79 (95% CI, 0.69–0.88)/0.90 (95% CI, 0.83–0.97).

**Conclusion:**

Frailty and pre‐frailty as assessed by FFP and SFQ are commonly found in advanced stage cancer patients. Both questionnaires have a strong correlation and are associated with all‐cause mortality in this population. Since the SFQ is easier and quicker to perform, it can be used remotely, and with untrained staff, it might facilitate earlier preventive measures and initiate further actions to mitigate its impact.

## 1 Introduction

The population with advanced cancer often suffers from comorbidities and is generally older [1, 2]. Common conditions linked to both advanced cancer and ageing include heart failure [3], neurological [4] and kidney disease [5], but also metabolic diseases such as obesity [6] or catabolic states such as cachexia [7], malnutrition [8] and sarcopenia [9]. Such diseases are often associated with a reduction of physical function and performance [10] requiring more frequent, intensive and specialized hospital care resulting in a loss of independence for patients [11]. This complex condition can be summarized under frailty, which is estimated to have a global prevalence of approximately 10% in community dwellers older than 65 years [12], increasing to 25%–50% in people older than 85 years [13]. A high prevalence is also present in selected populations with specific diseases or conditions, such as patients with cancer (10%–40%), end‐stage renal disease (~40%), advanced heart failure (~40%), Alzheimer's disease (~30%) or nursing home residents (~50%) [14, 15, 16, 17, 18]. Cancer as well as related anti‐cancer treatments (such as conventional chemotherapy, targeted therapy, hormonal therapy and immunotherapy) can significantly challenge the physiological reserve of patients [19, 20, 21, 22]. While cachexia is an adverse prognostic factor in patients with advanced cancer, the role of sarcopenia is still under discussion [10, 23, 24, 25]. Newer research has shown that advanced stage cancer patients frequently develop a new form of cardiac wasting‐associated cardiomyopathy with increased morbidity and mortality as well as reduced quality of life [26, 27, 28, 29]. In this context of wasting diseases, frailty is very important to consider. Research has demonstrated that physical rehabilitation and nutritional support can improve frailty [30, 31]. Recently, a study found that suboptimal nutritional status in men with metastatic castrate‐resistant prostate cancer is associated with poorer health‐related quality of life and reduced overall survival [32]. The authors suggested that further interventional studies focusing on nutritional therapy or prevention of frailty are warranted to improve outcomes in this patient population [32]. The use of anabolic agents, such as selective androgen receptor modulators (SARMs), testosterone replacement or other anabolic steroids, could potentially counteract muscle loss and reduced strength associated with frailty [33, 34]. However, the use of these agents is typically approached with caution due to concerns about safety, side effects and inconsistent evidence regarding their long‐term efficacy [33]. Iron supplementation is recognized as a potentially effective intervention in cases with iron deficiency or anaemia [35]. Restoring normal iron levels can improve muscle function, energy and overall vitality [35]. Iron supplementation is recommended when laboratory tests confirm a deficiency, though its benefits may vary depending on the underlying causes of frailty [35]. Nonetheless, we believe that prevention should be a central focus. In this regard, it is crucial to highlight the importance of preventive strategies that intervene before frailty is fully established. The transition from pre‐frailty to frailty represents a critical window of opportunity for implementing interventions that may slow or even prevent the progression of frailty. These strategies could include physical activity programmes, nutritional interventions and psychosocial support [31].

By including frailty assessments in daily clinical routine, clinicians are able to tailor treatment decisions to the specific needs of their patients [2]. ‘Frailty Index’ is a comprehensive tool, that includes multiple health indicators such as comorbidities, physical and cognitive status and functional ability [36]. It is widely used in various populations, including cancer patients, as it captures a broad spectrum of frailty dimensions [37, 38]. The ‘Comprehensive Geriatric Assessment’ includes a variety of tests, such as the ‘Mini‐Mental State Examination’, the ‘Timed Up and Go test’ and the ‘Activities of Daily Living scale’ [39]. It is often used in oncology to evaluate comprehensive frailty, including cognitive and functional aspects. The ‘Fried Frailty Phenotype’ (FFP) questionnaire, consisting of five components, is commonly used in geriatric oncology settings because of its focus on physical performance, which is particularly relevant for cancer patients undergoing treatment [40, 41]. Since these questionnaires take up a lot of time, simple, rapid screening versions are required in clinical practice. ‘Clinical Frailty Scale’ ranges from very fit to terminally ill and is easy to use, requiring only a clinical assessment [42]. It is particularly useful in oncology settings for identifying frailty in cancer patients and guiding treatment decisions [43, 44]. The Simple FRAIL Questionnaire (SFQ) also screens for frailty by exploring five domains and can be administered by health care professionals and caregivers [30]. As far as we know, the SFQ has not yet been validated in an oncology setting.

The aim of this study was to assess the prevalence of frailty in advanced cancer patients using the FFP as a standard method and SFQ as a novel method, to evaluate the correlation between these two questionnaires and to determine their ability to predict all‐cause mortality.

## 2 Methods

### 2.1 Study Population

From November 2017 to February 2020, we prospectively recruited 251 hospitalized cancer patients from the Department of Haematology and Oncology at Charité Medical School, Campus Benjamin Franklin, and the Department of Cardiology and Pneumology at the Medical University of Göttingen. Patients were screened upon admission. Only adult patients (age ≥ 18 years), with histologically confirmed cancer, were eligible for enrolment. Exclusion criteria included (i) another cancer diagnosis in the past 5 years; (ii) acute infection and/or antibiotic treatment; (iii) history of significant cardiovascular disease (e.g., history of heart failure, severe valvular disease, history of myocardial infarction or coronary artery disease); (iv) chronic obstructive pulmonary disease (COPD) GOLD stage > II (GOLD stages III–IV were accepted in lung cancer); and (v) missing data for one or more frailty questionnaires. Advanced stage cancer was defined as stage III/IV Union for International Cancer Control (UICC) [45], stage III/IV for Ann Arbor classification [46] and stage III for Durie and Salmon classification [47]. Main reasons for hospitalization were application of chemotherapy that required hospitalization (43%), diagnostic procedures (40%), general worsening of the clinical condition (10%), neurological symptoms (4%) or pain exacerbation (3%).

### 2.2 Study Design

All study participants underwent the same examination protocol at recruitment: a detailed collection of medical history, physical examination, blood sample and evaluation of frailty. We classified patients as malnourished if they had a BMI below 18.5 kg/m^2^ [48]. Iron deficiency was defined as (1) serum ferritin < 100 ng/mL or (2) when serum ferritin was 100–299 ng/mL and transferrin saturation < 20% [35]. The patients were followed up by regular interrogation of electronic hospital records and by telephone. All participants of the study signed a written informed consent. The study was approved by the local ethics committee and conforms to the declaration of Helsinki.

### 2.3 Fried Frailty Phenotype (FFP)

The FFP considers deficits in five domains: Shrinking, Weakness, Low physical activity, Poor endurance and Energy and Slowness [40]. The first three components were assessed through the respective self‐reported questions. ‘Endurance and energy’ were tested through hand‐grip‐test using Jamar hydraulic hand dynamometer on the dominant hand (defined as right‐or left‐handed). Three serial tests of maximum grip strength were obtained, and a mean value was determined. The assignment of FFP‐points was stratified by sex and body mass index (BMI) quartiles according to Hall et al. [49]. ‘Slowness’ was examined through a 4‐m‐gait speed test: Walking time of 4 m at a normal pace was recorded two times. For the assignment of FFP‐points, the best time was taken and stratified by sex and height according to Hall et al. The five domains are equally weighted. Three or more (maximum of 5) of the above‐mentioned criteria had to be present for an individual to be considered frail. Patients who obtained 1 or 2 points were pre‐frail and those with 0 points robust.

### 2.4 Simple FRAIL Questionnaire (SFQ)

The SFQ includes five self‐reported questions regarding Fatigue, Resistance, Ambulation, Illnesses and Loss of weight [30]. According to SFQ, frailty status was assigned to participants with a score of 3 points or greater. Subjects with 1–2 points were considered pre‐frail and those with 0 points robust, respectively.

### 2.5 Statistical Analysis

Normally distributed data are presented as mean ± standard deviation (SD) and non‐normally distributed data as median with interquartile range. We used the Kolmogorov–Smirnov test to assess normal distribution. Chi‐squared test was chosen for the analysis of the contingency tables and Fisher's Exact test if the tables contained at least one cell assignment under five. We used analysis of variance (ANOVA) with Fisher's post hoc test for normally distributed groups. Kruskal–Wallis test was used for non‐normally distributed data as appropriate. The correlation between FFP and SFQ was assessed using Spearman's rho correlation coefficient (*r*_*s*_). Hazard ratios (HRs) with a 95% confidence interval (CI) for risk factors and significance levels are given for results of Cox proportional hazards survival analyses. Kaplan–Meier cumulative survival curves for 24 months were constructed for illustrative purposes. The assumption of proportional hazard was formerly assessed using Schoenfeld residuals and the chi‐square goodness‐of‐fit tests. Prediction accuracy for the Cox proportional hazards models was evaluated by time‐dependent multivariable adjusted ROC curves and Uno's C. IBM Statistical Package for the Social Sciences (SPSS) 29, StataCorp. 2023. Stata Statistical Software: Release 18. College Station, TX: StataCorp LLC., and SAS/STAT software, Version 9.4 Copyright 2023 SAS Institute Inc. were used for statistical analysis. A *p* value < 0.05 was considered statistically significant in all analyses. Since this study was not designed as a confirmatory study, but to identify patterns, it was not necessary to make an adjustment for tests for multiplicity. The used significance tests therefore have a descriptive character.

## 3 Results

### 3.1 Study Population

The 251 cancer patients evaluated were 61 ± 13 years old, 53% were male, the mean BMI was 25.3 ± 4.8 kg/m^2^ and 78% were classified as advanced cancer stage. Further patients' characteristics are shown in Table 1A and cancer entities in Table S1.

**TABLE 1A:** Baseline characteristics.

Variables	Total cohort (*n* = 251)	Fried Frailty Phenotype Questionnaire				Simple Frail Questionnaire			
		Robust (*n* = 78, 31%)	Prefrail (*n* = 131, 52%)	Frail (*n* = 42, 17%)	*p*	Robust (*n* = 117, 47%)	Prefrail (*n* = 102, 40%)	Frail (*n* = 32, 13%)	*p*
Clinical characteristics									
Age	61 ± 13	59 ± 13^#^	61 ± 14	66 ± 12	**0.036**	59 ± 14^§^, °	63 ± 13°	65 ± 11	**0.0046**
Male sex (*n*, %)	133 (53)	44 (56)	70 (53)	19 (45)	0.50	66 (56)	56 (55)	11 (26)	0.076
BMI (kg/m^2^)	25.3 ± 4.8	26.6 ± 5.0^$^, ^#^	25.1 ± 4.7	23.6 ± 4.2	**0.0034**	25.8 ± 4.5°	25.5 ± 5.3°	23.0 ± 4.2	**0.013**
Cancer stage ≥ III (*n*, %)	196 (78)	55 (71)	101 (77)	40 (95)	**0.0070**	84 (72)	84 (82)	28 (88)	0.066
Cancer type: solid (*n*, %)	148 (59)	43 (55)	73 (56)	32 (76)	**0.045**	65 (56)	58 (57)	25 (78)	0.061
Laboratory parameters									
Haemoglobin (g/dL)	11.6 ± 2.2	12.5 ± 2.0^$^, ^#^	11.4 ± 2.0^#^	10.5 ± 2.2	**< 0.0001**	12.0 ± 2.1°	11.5 ± 2.1°	10.2 ± 1.8	**< 0.0001**
Leucocytes (× 10^9^/L)	7.5 ± 6.4	7.9 ± 6.6	7.5 ± 6.9	6.5 ± 4.0	0.49	7.8 ± 7.5	6.9 ± 3.6	8.1 ± 8.7	0.49
Platelets (× 10^9^/L)	253 ± 125	255 ± 120	254 ± 124	245 ± 139	0.92	253 ± 118	246 ± 113	276 ± 176	0.50
Sodium (mmol/L)	139 ± 4	139 ± 4	140 ± 3^#^	138 ± 5	**0.040**	140 ± 4	139 ± 3	138 ± 5	0.12
Potassium (mmol/L)	4.0 ± 0.5	4.0 ± 0.4	4.0 ± 0.5	3.8 ± 5	0.25	4.0 ± 0.5^§^, °	3.9 ± 0.5	3.8 ± 0.4	**0.017**
Creatinine (mg/dL)	0.85 ± 0.33	0.84 ± 0.21	0.83 ± 0.25	0.95 ± 6.0	0.11	0.83 ± 0.22°	0.82 ± 0.22°	1.03 ± 0.69	**0.0029**
eGFR (mL/min)	87 ± 21	89 ± 19	88 ± 20	81 ± 25	0.087	91 ± 20°	87 ± 19°	75 ± 28	**0.0009**
GOT (U/L)	28 (22–37)	29 (23–36)	26 (21–35)	33 (22–52)	0.13	28 (23–35)	28 (22–37)	30 (21–51)	0.71
GPT (U/L)	22 (15–36)	24 (16–40)	22 (15–35)	22 (12–33)	0.55	23 (15–40)	23 (15–34)	21 (14–38)	0.60
GGT (U/L)	49 (28–121)	43 (24–92)	50 (27–95)	71 (37–222)	**0.012**	43 (25–102)	53 (30–95)	87 (46–221)	**0.021**
Albumin (g/L)	37 ± 5	39 ± 4^$^, ^#^	37 ± 5^#^	34 ± 5	**< 0.001**	38 ± 5^§^, °	37 ± 5°	33 ± 5	**< 0.0001**
Secondary diagnoses									
Arterial hypertension (*n*, %)	102 (41)	32 (41)	52 (40)	18 (43)	0.93	47 (40)	43 (42)	12 (38)	0.89
Diabetes mellitus (*n*, %)	28 (11)	7 (9)	17 (13)	4 (10)	0.69	11 (9)	12 (12)	5 (16)	0.59
COPD (*n*, %)	10 (4)	4 (5)	5 (4)	1 (2)	0.78	3 (3)	6 (6)	1 (< 1)	0.44
Chronic kidney disease (*n*, %)	30 (12)	6 (8)	15 (12)	9 (21)	0.084	9 (8)	9 (9)	12 (38)	**< 0.0001**
Previous stroke (*n*, %)	13 (5)	2 (3)	3 (2)	2 (5)	0.68	1 (1)	5 (5)	1 (3)	0.14
Anaemia (*n*, %)	< 1%	48 (62)	47 (36)	13 (31)	**0.0003**	57 (49)	62 (61)	24 (75)	**0.017**
Peripheral vascular diseases (*n*, %)	10 (4)	32 (41)	52 (40)	18 (43)	0.93	47 (40)	43 (42)	12 (38)	0.89
Malnutrition (*n*, %)	12 (5)	2 (3)	6 (5)	4 (10)	0.23	4 (3)	5 (5)	3 (9)	0.35
Iron deficiency (*n*, %)	80 (32%)	26 (33)	47 (36)	7 (17)	0.063	45 (38)	28 (28)	7 (22)	0.94
Medication at study									
Acetylsalicylic acid (*n*, %)	21 (8)	5 (6)	9 (7)	7 (17)	0.10	8 (7)	8 (8)	5 (16)	0.27
ACE inhibitors or ARBs (*n*, %)	66 (26)	21 (27)	32 (24)	13 (31)	0.70	25 (21)	29 (28)	12 (38)	0.15
Calcium channel blockers (*n*, %)	42 (17)	14 (18)	20 (15)	8 (19)	0.80	14 (12)	20 (20)	8 (25)	0.13
Beta‐blockers (*n*, %)	53 (21)	18 (23)	26 (20)	9 (21)	0.86	25 (21)	18 (18)	10 (31)	0.26
Spironolactone (*n*, %)	4 (2)	1 (1)	2 (2)	1 (2)	0.82	0	2 (2)	2 (6)	**0.023**
Diuretics (*n*, %)	45 (18)	12 (15)	22 (17)	11 (26)	0.30	17 (15)	16 (16)	12 (38)	**0.0082**
Anticoagulants (*n*, %)	97 (39)	32 (41)	41 (31)	24 (57)	**0.0099**	37 (32)	41 (40)	19 (59)	**0.016**

*Note:* Values are means ± SD, *n* (%) or median and (IQR). *p* values are determined using the ANOVA, Kruskal–Wallis test or chi‐square test. *p* < 0.05 are bold.

Abbreviations: ACE, angiotensin converting enzyme inhibitors; ARBs, angiotensin II receptor blocker; BMI, body mass index; COPD, chronic obstructive disease; eGFR, estimated glomerular filtrate rate; GGT, γ‐glutamyltransferase; GOT, glutamate oxaloacetate transaminase; GPT, glutamate pyruvate transaminase.

^$^ *p* < 0.05 versus pre‐frail.

^#^ *p* < 0.05 versus frail.

^§^ *p* < 0.05 versus pre‐frail.

° *p* < 0.05 versus frail.

### 3.2 Frailty Questionnaires

**FIGURE 1:**
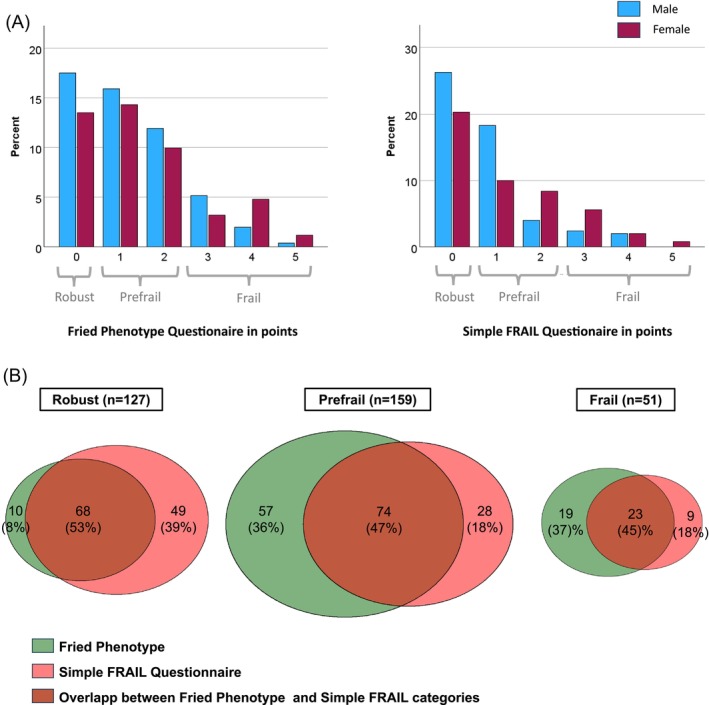
(A) Fried Phenotype and the Simple FRAIL Questionnaire responses in points separated by sex. (B) Venn diagram with the distribution of cancer patients in the categories Robust/Prefrail/Frail according to the Fried Phenotype and the Simple FRAIL Questionnaire (*n* = 251).

We found that the examiners needed about 10 min with the FFP questionnaire and 1–2 min with the SFQ. Using the FFP, Shrinking, Poor endurance and energy, Low physical activity, Weakness and Slowness were present in 111 (44%), 66 (26%), 45 (18%), 60 (24%) and 55 (22%) of cancer patients. Using the SFQ, Fatigue, Resistance, Ambulation, Illnesses and Weight loss were present in 36 (14%), 25 (10%), 62 (25%), 28 (11%) and 92 (37%) cancer patients. According to the FFP, 78 (31%) cancer patients were pre‐frail, and 42 (17%) were frail. Using the SFQ, 102 (41%) cancer patients were identified as pre‐frail and 32 (13%) as frail (Figure 1A,B). In both assessments, those who were frail were older, more frequently had a solid cancer and their BMI was significantly lower (Table 1A and Table 1B). The correlation between both frailty scores was strong (*r*_*s*_ = 0.65, *p* < 0.001).

**TABLE 1B:** Frailty assessments.

Fried Frailty Phenotype Questionnaire	Total cohort (*n* = 251)	Robust (*n* = 78, 31%)	Prefrail (*n* = 131, 52%)	Frail (*n* = 42, 17%)	*p*
Score (pts.)	1.3 ± 1.3	0.0 ± 0.0^$^, ^#^	1.4 ± 0.5^#^	3.6 ± 0.5	< 0.0001
Shrinking (*n*, %)	111 (44)	0	79 (60)	32 (76)	< 0.0001
Poor endurance (*n*, %)	66 (26)	0	37 (28)	29 (69)	< 0.0001
Low physical activity (*n*, %)	45 (18)	0	13 (10)	32 (76)	< 0.0001
Weakness (*n*, %)	60 (24)	0	34 (28)	25 (60)	< 0.0001
Slow walking (*n*, %)	55 (22)	0	23 (18)	32 (76)	< 0.0001

Simple Frail Questionnaire	Total cohort (*n* = 251)	Robust (*n* = 117, 47%)	Prefrail (*n* = 102, 40%)	Frail (*n* = 32, 13%)	*p*
Score (pts.)	1.0 ± 1.2	0.0 ± 0.0^§^, °	1.3 ± 0.5°	3.4 ± 0.6	< 0.0001
Fatigue (*n*, %)	36 (14)	0	17 (17)	19 (59)	< 0.0001
Resistance (*n*, %)	25 (10)	0	6 (6)	19 (59)	< 0.0001
Ambulation (*n*, %)	62 (25)	0	30 (29)	32 (100)	< 0.0001
Illnesses (*n*, %)	28 (11)	0	14 (14)	14 (44)	< 0.0001
> 5% loss of weight in 6 months (*n*, %)	92 (37)	0	66 (65)	26 (81)	< 0.0001

*Note:* Values are means ± SD, *n* (%) or median and (IQR). *p* values are determined using the ANOVA or chi‐square test.

^$^ *p* < 0.05 versus pre‐frail.

^#^ *p* < 0.05 versus frail.

^§^ *p* < 0.05 versus pre‐frail.

° *p* < 0.05 versus frail.

### 3.3 Predictors of Survival

**FIGURE 2:**
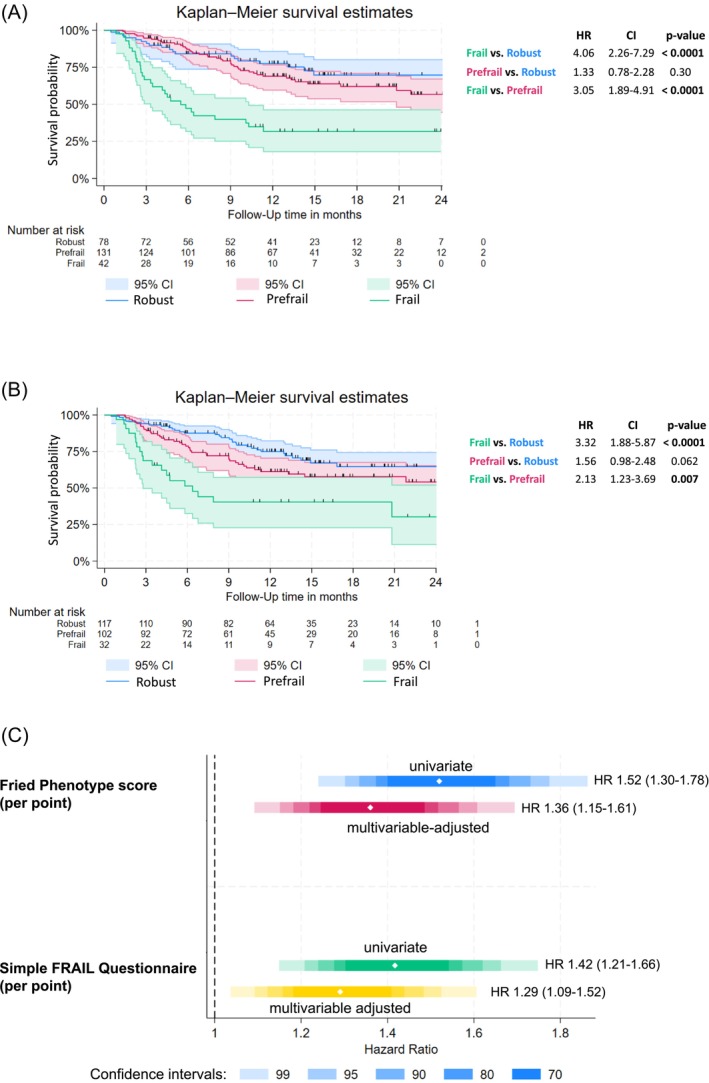
(A) Cumulative survival of all cancer patients (*n* = 251) according to the Fried Phenotype. (B) Cumulative survival of all cancer patients (*n* = 251) according to the Simple FRAIL Questionnaire. (C) Comparison of hazard ratios including confidence intervals from survival analysis according to the Fried Phenotype and the Simple FRAIL Questionnaire (*n* = 251). *Multivariable adjusted for age, cancer stage and type, anti‐cancer therapy naïve, sex, body mass index, chronic kidney disease and anaemia.

Cancer patients were followed for a mean of 11.6 months (maximum 27 months), and 91 (36%) patients died during follow‐up. Using univariable and multivariable Cox‐proportional hazard analysis, adjusted for other significant predictors of mortality as well as clinically relevant parameters, we found that both frailty questionnaires were independent predictors of mortality (Tables 2A and 2B). Both multivariable survival models meet the proportional hazard assumption and Bayesian information criterion (BIC) between both models were approximately similar. In Figure 2A,B, we show that especially cancer patients who were classified as frail had the worst survival. The median survival of the frail group for FFP was 5.5 months, 95% CI [3.3–10.3], and for SFQ was 6.4 months, 95% CI [2.8–24.0]. For FFP and SFQ, the median survival was not reached for the prefrail and robust groups. The FFP and SFQ per point show similar univariable and multivariable‐adjusted hazard ratios for mortality (Figure 2C).

**TABLE 2A:** Multivariable Cox proportional hazard analyses for mortality in cancer patients in relation to the Fried Phenotype score (*n* = 251).

	Univariable model			Multivariable model^a^ BIC: 897.36, global SRT: 0.54			
	HR	95% CI	*p*	HR	95% CI	*p*	SRT
Fried Frailty Phenotype score (per point)	1.52	1.30–1.78	< 0.001	1.36	1.15–1.61	< 0.001	0.42
Age (per year)	1.02	1.00–1.04	0.018	1.01	0.99–1.03	0.38	0.91
Cancer stage ≥ III vs. I/II	7.93	2.91–21.63	< 0.001	3.72	1.32–10.47	0.013	0.29
Cancer type (solid vs. haematologic)	4.36	2.54–7.48	< 0.001	2.68	1.50–4.79	0.001	0.66
Anticancer therapy‐naive (yes vs. no)	0.15	0.06–0.41	< 0.001	0.29	0.10–0.82	0.020	0.20
Sex (female vs. male)	0.96	0.64–1.45	0.85	0.91	0.59–1.39	0.65	0.65
Body mass index (per kg/m^2^)	0.96	0.92–1.01	0.11	0.97	0.93–1.02	0.26	0.12
Chronic kidney disease (yes vs. no)	1.43	0.45–4.51	0.55	0.81	0.25–2.59	0.73	0.56
Anaemia (yes vs. no)	1.32	0.87–2.02	0.20	1.06	0.68–1.65	0.79	0.98

Abbreviations: BIC, Bayesian information criterion; SRT, *p* value for Schoenfeld residual test.

^a^ Multivariable adjusted for age, cancer stage and type, anti‐cancer therapy naïve, sex, body mass index, chronic kidney disease and anaemia.

**TABLE 2B:** Multivariable Cox proportional hazard analyses for mortality in cancer patients in relation to the Simple FRAIL score (*n* = 251).

	Univariable model			Multivariable model^a^ BIC: 901.42, global SRT: 0.54			
	HR	95% CI	*p*	HR	95% CI	*p*	SRT
Simple Frail score (per point)	1.42	1.21–1.66	< 0.001	1.29	1.09–1.52	0.003	0.41
Age (per year)	1.02	1.00–1.04	0.018	1.01	0.99–1.03	0.40	0.83
Cancer stage ≥ III vs. I/II	7.93	2.91–21.63	< 0.001	4.22	1.50–11.83	0.006	0.26
Cancer type (solid vs. haematologic)	4.36	2.54–7.48	< 0.001	2.70	1.50–4.84	0.001	0.41
Anticancer therapy‐naive (yes vs. no)	0.15	0.06–0.41	< 0.001	0.29	0.10–0.83	0.021	0.29
Sex (female vs. male)	0.96	0.64–1.45	0.85	0.80	0.52–1.24	0.32	0.75
Body mass index (per kg/m^2^)	0.96	0.92–1.01	0.11	0.97	0.93–1.02	0.27	0.13
Chronic kidney disease (yes vs. no)	1.43	0.45–4.51	0.55	0.93	0.29–2.96	0.90	0.50
Anaemia (yes vs. no)	1.32	0.87–2.02	0.20	1.13	0.72–1.75	0.59	0.83

Abbreviations: BIC, Bayesian information criterion; SRT, *p* value for Schoenfeld residual test.

^a^ Multivariable adjusted for age, cancer stage and type, anti‐cancer therapy naïve, sex, body mass index, chronic kidney disease and anaemia.

### 3.4 Prediction Accuracy and Correlation of Frailty Status Identified With FFP and SFQ

**FIGURE 3:**
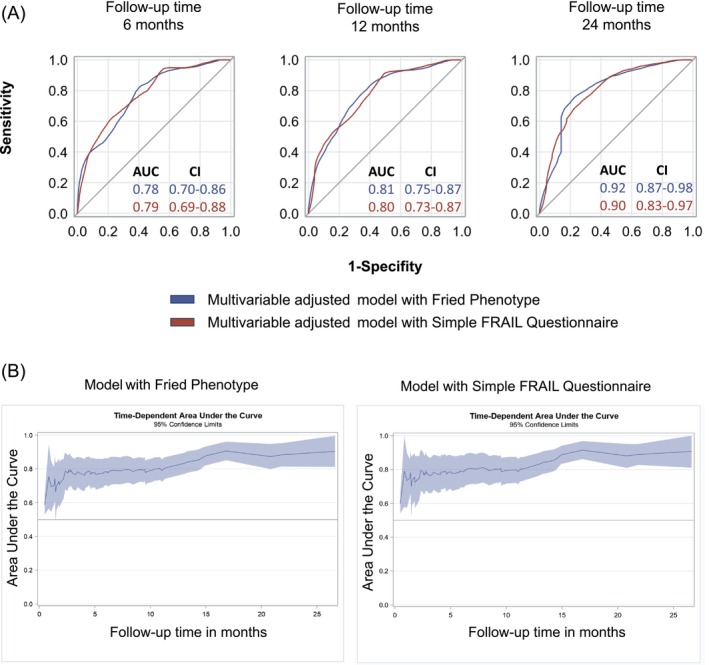
(A) Time‐dependent ROC curves according to the Fried Phenotype and the Simple FRAIL Questionnaire (*n* = 251). *Multivariable adjusted for age, cancer stage and type, anti‐cancer therapy naïve, sex, body mass index, chronic kidney disease and anaemia. (B) Time‐dependent area under the curve according to the Fried Phenotype and the Simple FRAIL Questionnaire (*n* = 251). *Multivariable adjusted for age, cancer stage and type, anti‐cancer therapy naïve, sex, body mass index, chronic kidney disease and anaemia.

The time‐dependent multivariable adjusted ROC curves and the corresponding area under the receiver operating characteristic curve for 6, 12, 18 and 24 months of follow‐up for FFP and SFQ are shown in Figure 3A and Table 2C and are similar between FFP and SFQ. The comparison of the multivariable‐adjusted area under the curve over the entire observation period for the FFP and the SFQ shows similar curves for both questionnaires (Figure 3B). Uno's C is 0.76 for the multivariable‐adjusted FFP and the SFQ, and there is no significant difference between the scores (difference = −0.001, *p* = 0.894, Tables 2A, 2B–2C).

**TABLE 2C:** Multivariable adjusted, time‐dependent area under the curve for the Fried Frailty Phenotype and the Simple Frail Questionnaire and, in addition, comparison of the concordance statistic.

	Fried Frailty Phenotype score^a^			Simple Frail score^a^			Differences in Uno's concordance statistic	
	AUC	95% CI	Uno's C (SE)	AUC	95% CI	Uno's C (SE)	Estimate	*p* value
6‐month follow‐up	0.78	0.70–0.86	0.760 (0.031)	0.79	0.69–0.88	0.761 (0.045)	−0.001	0.894
12‐month follow‐up	0.81	0.75–0.87		0.80	0.73–0.87			
18‐month follow‐up	0.91	0.85–0.97		0.91	0.85–0.97			
24‐month follow‐up	0.92	0.87–0.98		0.90	0.83–0.97			

Abbreviation: Uno's C, Uno's concordance statistic.

^a^ Multivariable adjusted for age, cancer stage and type, anti‐cancer therapy naïve, sex, body mass index, chronic kidney disease and anaemia.

## 4 Discussion

The main finding from this study is that frailty and pre‐frailty as assessed by FFP and SFQ are frequently seen in patients with mostly advanced cancer, both questionnaires correlate well with good internal consistency and both independently predict all‐cause mortality to a similar extent. This study shows that including frailty assessment in clinical practice can identify those patients that might need extra help and attention by caregivers and physicians.

In the literature, only a limited number of studies used different frailty screening tools in the same cohort and compared the different outcomes [50, 51, 52]. While the FFP questionnaire has often been used in cancer patients to assess frailty and has been shown to be associated with increased mortality, morbidity and decreased quality of life [53, 54], the SFQ questionnaire has predominantly been used to assess frailty in the general adult and elderly population [55, 56, 57]. To the best of our knowledge, this was the first study to compare the FFP and SFQ questionnaire in a cohort of mostly advanced stage cancer patients. It is essential to acknowledge that the FFP questionnaire, while comprehensive in its assessment, incorporates physical performance tests, thus necessitating a more considerable time investment for its completion compared to the SFQ that is based on five self‐reported questions. Our investigation revealed a substantial contrast in administration times, with the FFP demanding an average of approximately 10 min per patient within this cohort. In contrast, the SFQ exhibited efficiency, requiring only 1–2 min per patient for completion. Given the complexities of the medical conditions, time efficiency becomes a critical factor in clinical practice. Furthermore, the SFQ can be performed by any caregiver or member of the medical team without prior training, while for the FFP medical staff first needs to be trained to correctly administer a 4‐m walking test and hand grip strength test—additionally requiring a hand dynamometer. Since the SFQ only depends on self‐reported questions, it can even be remotely administered through telephone contact with patients, making it a valuable screening tool to detect cancer patients in need of specialized and targeted care during telephone follow‐up of patients.

In our study, we found according to both scores that 13%–17% of patients were identified as frail and 41%–52% as pre‐frail. These frequencies are in line with other studies that have looked at cancer patients with similar disease stage, entities, age and sex [18]. This consistency across studies underscores the robustness of our results and their applicability within the broader context of cancer‐related frailty. Further, we observed a clear, but not complete, overlap in all categories and FFP recognized a higher proportion of frail/prefrail patients than SFQ despite strong correlation between both questionnaires. FFP is more comprehensive as it addresses more domains that may have an impact on presence of frailty. SFQ should be used as a screening tool as it relies on patients' subjective and after identifying of persons with physical frailty or at risk of frailty further examinations are needed to assess muscle strengths and physical status. Patients categorized as frail warrant comprehensive strategies that address their unique vulnerabilities, ultimately enhancing their quality of life and overall well‐being. However, a noteworthy aspect pertains to the patients classified as pre‐frail—a group characterized by a greater susceptibility to transition into frailty [58]. Recognizing this, it is vital to underscore the significance of preventive strategies that intervene before the full development of frailty. The trajectory from pre‐frailty to frailty represents a crucial window of opportunity to implement interventions that could potentially stall or mitigate the progression of frailty. Such strategies are multimodal and may encompass physical activity regimens (resistance and aerobic), nutritional interventions (caloric, protein or vitamin support), reduction of polypharmacy and psychosocial support [31]. In addition, all persons older than 70 should be screened for frailty. By investing in these preventive measures, we can potentially alter the course of frailty development and contribute to improved outcomes for individuals on the cusp of experiencing heightened vulnerability. In essence, our study accentuates the value of early identification, not solely for addressing the needs of frail patients but also for devising proactive measures to support those at the precipice of frailty. By emphasizing personalized care and preventive strategies, healthcare practitioners can play a pivotal role in enhancing the lives of both frail and pre‐frail individuals, ultimately fostering a more resilient patient population.

Consideration of longitudinal studies is warranted to comprehensively explore the lasting effects of preventive interventions on the development of frailty and associated clinical outcomes over longer periods of time. A thorough examination of the cost‐effectiveness profiles attributed to different modalities for assessing frailty and to different interventions is an important avenue. These considerations could lead to nuanced insights that are important for health care decision makers. At the same time, careful evaluation of the pragmatic feasibility of frailty assessment measures in authentic clinical settings is a mandatory pursuit. Such evaluation is central to achieving the seamless integration of frailty management paradigms into the structure of standard patient care protocols. Conducting these empirical investigations will further advance our iterative refinement of the framework underlying frailty assessment and intervention paradigms. This iterative refinement, in turn, holds the potential to create a transformative path forward that will deliver expanded care paradigms and significantly improved outcomes for people who are on the precipice of frailty vulnerability [59].

## 5 Limitations

As a limitation, there may be additional confounders that have an impact on frailty such as anti‐cancer therapy and other clinical comorbidities. Since 81% of our cancer patients had already received some kind of anti‐cancer therapy before the time of assessment, it has to be acknowledged that the patients in this cohort should be considered a high‐risk group for frailty. However, we included hospitalized cancer patients with different cancer entities—representing a real‐world scenario of hospitalized cancer patients. As an association between frailty and cardiac disease has been previously reported [59], we included only patients with cancer without any significant cardiac disease or acute infection to minimize risks of bias. Another bias might be the fact that most of the patients had an advanced disease stage (78%) with a high mortality burden overall (median survival 11 months). Thus, we used all‐cause mortality as the outcome measure, an unbiased metric. This was done since specific cause of death assessment is very complicated in cancer patients—with autopsies rarely being performed in these patients and cancer patients often dying at home or in specialized hospice care where exact cause of death determination is very complicated [60, 61]. At the same time, in future studies, it would be of interest to test whether frailty can affect specific causes of death.

## 6 Conclusions

Frailty and pre‐frailty are common among hospitalized cancer patients and are independently associated with all‐cause mortality. Given that the Simple FRAIL Questionnaire is quick and easy to administer, can be used remotely and requires no specialized training for staff, it may be more practical for clinical use than the Fried Frailty Phenotype. By improving frailty detection, the Simple FRAIL Questionnaire could facilitate earlier preventive measures and enhance interventions to mitigate its impact.

## Conflicts of Interest

U.W. is supported by a Clinical Fellowship Grant from the Berlin Institute of Health and has received speaker fees and/or contributions to congresses from Abbott, AstraZeneca, Bayer, Berlin Chemie, Bristol Myers Squibb, GE Healthcare, Pfizer, Philips and Servier, all outside the submitted work. D.P.M.: speaker fees and honoraria: Servier, Amgen, Merck, Sanofi, MSD, AstraZeneca, Pierre Fabre, GSK, Seagen, G1, Onkowissen, COR2ED, Taiho, Takeda, Incyte, Cureteq, 21up, Medscape, Aptitude Health, Regeneron. Funding (inst): Servier, Amgen. L.B. has received honoraria from AbbVie, Amgen, Astellas, Bristol Myers Squibb, Celgene, Daiichi‐Sankyo, Gilead, Hexal, Janssen, Jazz Pharmaceuticals, Menarini, Novartis, Pfizer, Roche, Sanofi and Seattle Genetics and has received research support from Bayer and Jazz Pharmaceuticals. U.K. has served on advisory boards for Roche, Janssen‐Cilag, Celgene, Takeda, Bristol Myers Squibb, Gilead, Hexal, Pfizer, AstraZeneca and Pentixapharm, has received clinical research support from Janssen‐Cilag, Novartis, Takeda, Bristol Myers Squibb, Roche and Pfizer and has received travel support from Roche, Bristol Myers Squibb, Gilead, Takeda, Janssen‐Cilag and Celgene. M.K. is supported by a Clinician Scientist Professorship Grant from the Else Kroener‐Fresenius‐Foundation and has received personal fees and grant support from Daiichi‐Sankyo, Adrenomed, Sphingotec and Vifor Pharma, all outside the submitted work, and is a part‐time employee of 4TEEN4 Pharmaceuticals. U.L. has received institutional research grants from Amgen, Bayer and Novartis and has received speaker or consulting honoraria from AstraZeneca, Bayer, Boehringer, Amgen, Sanofi, Novartis and Novo Nordisk. S.v.H. has been a paid consultant for and/or received honoraria payments from AstraZeneca, Bayer, Boehringer Ingelheim, BRAHMS, Chugai, Grünenthal, Helsinn, Hexal, Novartis, Pharmacosmos, Respicardia, Roche, Servier, Sorin and Vifor. He also reports research support from Amgen, AstraZeneca, Boehringer Ingelheim, Innovative Medicines Initiative (IMI) and the German Center for Cardiovascular Research (DZHK). All other authors have reported that they have no relationships relevant to the contents of this paper to disclose.

## Supporting information


**Table S1:** Cancer entities.
