# Supplementary material for: The Prognostic Role of Frailty and Its Recognition With Simple FRAIL and Fried Frailty Questionnaires in Advanced Cancer Patients
Source: J Cachexia Sarcopenia Muscle. 2025 Oct 12;16(5):e70076. doi: 10.1002/jcsm.70076 (PMC12515984; doi:10.1002/jcsm.70076)
Supplement: Supplementary file 1 — Table S1: Cancer entities. [file JCSM-16-e70076-s001.docx]

Supplementary Table S1: Cancer entities

| Cancer entity (n=251) | Solid / hematologic cancer | n, % |
| --- | --- | --- |
| Colorectal cancer | Solid | 26 (11) |
| Breast cancer | Solid | 25 (10) |
| Non-small cell lung cancer | Solid | 18 (7) |
| Pancreatic cancer | Solid | 17 (3) |
| Oral cavity and salivary glands cancer | Solid | 13 (5) |
| Malignant melanoma of choroid | Solid | 7 (3) |
| Gynaecologic cancer  (vulva, cervix, fallopian tube, ovary, uterus) | Solid | 4 (2) |
| Small-cell-lung-cancer | Solid | 6 (2) |
| Oesophagus | Solid | 5 (2) |
| Hepatic-biliary tract cancer | Solid | 5 (2) |
| Stomach | Solid | 4 (2) |
| Soft tissue sarcoma | Solid | 3 (1) |
| Prostatic cancer | Solid | 3 (1) |
| Other cancer (brain, thyroid gland, kidney, urogenital, testicular, malignant melanoma of the skin, urothelial, thymus, peritoneal mesothelioma, cancer of unknow primary) | Solid | 12 (4) |
| Non-Hodgkin lymphoma | Hematological | 71 (28) |
| Classic Hodgkin lymphoma | Hematological | 21 (8) |
| Multiple myeloma | Hematological | 11 (4) |
